# Posture Status Differences Between Preschool Boys and Girls

**DOI:** 10.3390/jfmk10020101

**Published:** 2025-03-24

**Authors:** Anida Kapo-Gurda, Amin Efendić, Indira Mahmutović, Siniša Kovač, Husnija Kajmović, Safet Kapo, Jožef Šimenko

**Affiliations:** 1 Faculty of Education, University of Sarajevo, 71000 Sarajevo, Bosnia and Herzegovina; imahmutovic@pf.unsa.ba; 2 Faculty of Sports and Physical Education, University of Sarajevo, 71000 Sarajevo, Bosnia and Herzegovina; aminefendic@hotmail.com (A.E.); sinisa.kovac@fasto.unsa.ba (S.K.); husnija.kajmovic@fasto.unsa.ba (H.K.); safet.kapo@fasto.unsa.ba (S.K.); 3 Faculty of Sport, University of Ljubljana, 1000 Ljubljana, Slovenia; jozef.simenko@fsp.uni-lj.si

**Keywords:** assessment, body posture, deformities, early childhood, gender differences

## Abstract

**Background/Objectives**: The preschool period plays an essential role in shaping a child’s overall development, which influences physical, emotional, social, and cognitive growth. At this stage, establishing proper postural habits is essential, as it can have lasting effects on health, well-being, helps to prevent future issues, and supports overall development. Therefore, the present work aims to determine the differences in postural status between boys and girls of preschool age. **Methods**: The sample of participants consisted of 92 children (n = 46 boys and n = 46 girls); the average age for girls was 5.41 ± 0.30 years and for boys it was 5.53 ± 0.31 years. Data were collected using licensed state-of-the-art diagnostic equipment, Contemplas 3D Posture Compact, using 16 variables to assess postural status with a Mann–Whitney U test. **Results**: The results of this study indicate that boys have more pronounced deformities in the following variables: shoulder displacement (*p* = 0.047), pelvic obliquity (*p* = 0.000), sag. distance cervical spine–sacrum (*p* = 0.029), sag. distance thoracic spine–sacrum (SDTS) (*p* = 0.016), and sag. distance lumbar spine–sacrum (SDLS) (*p* = 0.005). **Conclusions**: This study confirmed gender differences in postural characteristics in preschool children. Boys showed a greater tendency towards postural deviations, indicating the necessity for specific interventions and programs to improve their posture. On the basis of the results of this research, it is recommended to carry out cross-cultural research that would enable the comparison of results among children from different environments and cultural contexts in order to determine possible differences and particularities in the development of postural characteristics. Future research should include larger and more diverse samples of participants, including children from rural and urban areas, in order to ensure the representativeness and generalizability of the results. In addition, conducting a longitudinal study that would monitor the postural characteristics of children through different developmental stages is suggested, aiming to identify critical periods for intervention and to determine, more precisely, development trends within the context of gender differences.

## 1. Introduction

The preschool period encompasses the period from the third year until the beginning of primary schooling (five to seven years) [1]. During that period, a child masters various activities, processes a large amount of different information, develops his speech to such a level that brings him close to the speech of adults, begins to think logically, and develops his senses [2]. The important aspects of child development at this age is the development of postural habits and patterns that can have a lifelong impact on the health and well-being of a child. In recent years, abnormalities related to body posture, often called postural defects, have increasingly been diagnosed in children [3,4,5,6,7]. In writing on this topic, many authors have tried to define what posture, in fact, is. Posture is considered a descriptive term for the relative position of body segments during rest or physical activity. Therefore, good posture implies an optimal relation between the reduction in load on the spinal column and the reduction in muscle work [8], i.e., the musculoskeletal balance that protects from the formation and gradual development of postural disorders in the structures responsible for keeping the body upright or stable, whether during movement or at rest [9,10]. On the other hand, researchers describe the postural status as a quality interrelationship of individual body segments aiming at maintaining the correct and upright body position without disturbing its stable position or falling [11]. Therefore, it is considered that each motor task is performed successfully only if there is no significant disturbance of body stability (equilibrium position).

Correct body posture implies correct relations between all body segments. This should be especially emphasized when it comes to children of preschool age because that period of development is of crucial importance. Given that postural control undergoes significant transitions between ages 4 and 6 [12], this study aims to assess whether early deviations can already be observed in this developmental phase. It starts with establishing control of the head, then the torso, and finally achieving postural stability while standing.

The sensorimotor system, which controls postural stability, undergoes significant changes between the ages of four and six, reaching full maturity between the ages of seven and ten [13]. Because of the sensitivity of a child’s body, establishing proper posture is critically important during the preschool years and the early stages of schooling [14]. In this period, children begin to adopt basic patterns of movement and body posture. Correct posture is not only an aesthetic issue but also has important functional and health implications.

Primary health care emphasizes the importance of preventing, diagnosing, and treating postural deformities in children early. Identifying postural disorders is extremely important, especially at the preschool age, as poor posture can indicate serious health issues if left uncorrected. However, these problems are often overlooked [15,16,17,18]. The reason lies in the necessity of the early formation of a “pattern of proper body posture”, which, if created in early childhood, not only contributes to the proper growth and development of children but also has a positive impact on their health and quality of life [19]. Poor body posture in children of preschool and school age is an indicator of health problems that can become very serious if not corrected in time [20]. Nevertheless, these issues often go unnoticed until it is too late. This highlights the importance of educators’ role in supporting healthy growth and development. By consistently monitoring and periodically evaluating children’s posture using 3D analysis models, many health problems can be identified early—before they become serious. The Contemplas Templo 3D apparatus [18,21] is one of these methods, which enables the objective and detailed measurement of body position, i.e., postural status, by analysis in three-dimensional space. Contemplas 3D technology is a modern method that combines optical analysis, computer algorithms, and specialized software to obtain precise data on body posture. The method is non-invasive and identifies asymmetries in postural segments (head, shoulders, torso, pelvis) and changes in the curvature of the spine, such as kyphosis, lordosis, or scoliosis, with clear reference values and visual interpretation. Unlike traditional postural assessment methods, 3D motion capture provides quantifiable and objective data, reducing observer bias and improving reproducibility [20].

However, the results of previous research are inconsistent regarding the dynamics of the development of sagittal curves of the spinal column and the identification of critical periods regarding the postural status of children and adolescents [13,18,21,22,23,24]. Common to all these studies is their recognition of specific age-related dynamics. Moreover, one key finding is the identification of patterns in upright posture development, showing that as children grow, changes generally lead to reduced spinal mobility and more pronounced deviations from correct posture. Therefore, the present study aimed to test these assumptions with the main aim of discovering the differences in postural status between boys and girls aged 5 to 6 in the Sarajevo Canton area.

## 2. Materials and Methods

### 2.1. The Sample of Participants

The present study is of a descriptive cross-sectional design, where the sample of participants consisted of 92 children, of which n = 46 girls (5.41 ± 0.30 years; 115.82 ± 5.61 cm, 21.05 ± 3.37 kg (body mass); 16.74 ± 2.19 kg (fat-free mass; 15.84 ± 2.09 kg muscle mass)) and n = 46 boys (5.53 ± 0.31 years; 119.29 ± 5.39 cm, 23.44 ± 5.12 kg (body mass); 17.58 ± 2.93 kg (fat-free mass); 16.53 ± 2.78 kg (muscle mass)) from the Public Institution “Children of Sarajevo”, from the area of Sarajevo Canton. The sample was selected using a simple random sampling method, by which an equal share of boys and girls was achieved. The G*Power software (Version 3.1.9.7, Universität Kiel, Kiel, Germany) was used to determine the number of participants through a priori power analysis for an independent T-test [25]. The results were taken by the following assumptions: two-tailed test, with significance level set at α = 0.05, study power set at 0.80, effect size set at 0.3, and distribution ratio set at 1 [21]. The recommended total number of samples was 82 (41 per group). However, we recruited 46 participants per group, resulting in an actual study power of 0.95. Considering that all the children included in the research were minors, their parents/guardians signed a written consent form regarding the participation of their children in the research. The participants had no associated diseases from the spectrum of possible influence on postural disorders/deformations.

All procedures were carried out in accordance with recommendations of the Helsinki Declaration and ethical standards of the Ethics Committee of the University of Sarajevo. Additionally, the study received an ethical committee approval from the Ministry of Education, Science, and Youth of Sarajevo Canton No. 11/03-34-37155/20.

### 2.2. Testing Protocol

#### 2.2.1. Anthropometry

Body height was measured with an anthropometer, according to Martin. During the measurement, the subjects were barefoot and in underwear (girls in undershirts) and stood in an upright position on a firm horizontal surface. The head of the subject was in the horizontal Frankfort plane. The examiner stood on the left side of the subject and controlled whether the anthropometer device was placed vertically and directly along the back of the body. The horizontal arm of the anthropometer is lowered to the top of the head (vertex point) and supported firmly but without pressure. The measurement result for body height values was read with an accuracy of 0.1 cm. An experienced examiner performed the measurements.

#### 2.2.2. Body Composition

Body composition was measured using a Tanita BC-420MA digital scale (Tanita BC 420 MA Segmental Body Composition Analyzer, Tanita Corp., Tokyo, Japan, 2015) [26].

The scale’s measuring platform features four electrode plates on which the subject stands barefoot, wearing only underwear. A low-strength direct current is transmitted through these contact plates to the body, and the scale measures the body’s total electrical resistance. Using the integrated software, along with inputted data such as body height, age, and sex, the device calculated the following variables: body mass, muscle mass, and fat-free mass. The results for body composition are displayed with a precision of 0.1 kg and 0.1%.

#### 2.2.3. Posture Assessment

**Figure 1:**
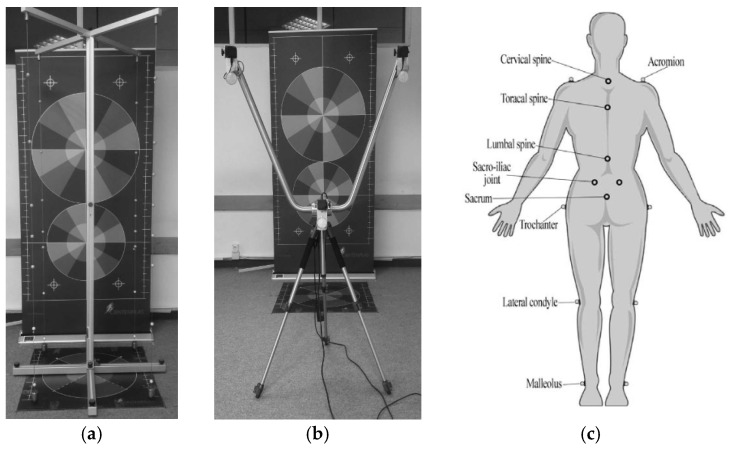
The Contemplas Templo apparatus ((**a**)—calibration frame; (**b**)—V camera frame; (**c**)—marker positions for 3D posture Compact protocol) [29].

The CONTEMPLAS Templo professional motion analysis software is a reliable and modern tool for diagnosing and detecting postural disorders. Due to its 3D analysis, Contemplas provides much more data on postural status compared to previous methods used to diagnose this problem. The validity of the system was confirmed by the Sports University of Cologne with an acceptable to excellent ICC (ICC > 0.70) agreement to the 3D Vicon system [27], while the inter and intra-rater reliability was also reported with acceptable to excellent consistency (ICC > 0.75) [22]. Such a method provides a wide range of possibilities for many specific needs and may be adapted to many hardware solutions with the help of which various analyses of movement and passive positions can be performed, resulting in conclusions about needs, knowledge, or cause of deformity [10]. 3D Compact is an analysis for assessing the postural status and status of the legs using three cameras that synchronically record and analyze the body from three perspectives simultaneously. In the 3D analysis, retroreflective marker balls were used (Figure 1b) [28].

Postural status in a standing position involves testing postural characteristics using 3D Contemplas software [27]. The procedure involves projecting the position of specific points on the body, after which a 3D kinematic model is created in the frontal plane. The CONTEMPLAS GmbH TEMPLO photometric apparatus, which includes a camera system and software analysis, by which it determines the position of marked points in space according to the 3D protocol, detects the positions of body segments in the calibrated space [22]. The measuring plate is placed on a flat surface. After determining the flat surface and placing the measuring plate, the Contemplas 3D posture compact mode is placed and fixed on the surface to avoid movement during the children’s positioning and additional space calibration. The 3D calibrator is placed on the surface with fluorescent markers. The 3D calibrator must be placed exactly in the center of the measuring plate, and the upper and lower beams, together with the vertical beam, must be ideally aligned and leveled (Figure 1a). The next step is to install a “V” frame that supports three cameras, which enable 3D analysis (Figure 1c). The camera’s distance from the center of the measuring plate must be at least 2 m and 15 cm. The images captured by the camera must be sharpened in software programming to begin space calibration. After completing the calibration, the 3D calibrator is packed away and testing can begin. An experienced examiner placed 16 reflective markers on the body of the subject, who only wore underwear, on specific points on the body: acromion (left and right), cervical spine, thoracic spine (kyphosis), lumbar spine (lordosis), crista iliaca posterior superior (left and right), sacrum, trochanter major (left and right), condylus laterallis (left and right), and malleolus laterallis (left and right), after which the subject is positioned in calibrated space with the back turned to the cameras, feet parallel and hip-width apart, and in the frontal plane. After that, the subject is instructed to take an upright position, look straight ahead, and relax his arms next to his body. Then, the projection takes place for 12 s, and photos are taken using three cameras (Basler acA645-100 gm/gc). The subject is photographed in an upright position. After the test, the markers were removed from the subject and placed on the next subject to be tested. The procedure of assembling and testing the instrument calibration is repeated whenever the place of testing is changed [22].

Four experienced certified measurers from the Sports Institute, with more than 7 years of experience working with the equipment, carried out the complete measurement procedure. To avoid subjectivity, only one person placed reflective markers in accordance with the protocol.

1. Sample of variables

The variables used for the purpose of this research provide basic information about the status of body position. The sample variables consist of 16 parameters. Shoulder displacement (SD) measures lateral shoulder asymmetry in the frontal plane; pelvic obliquity (PO) displays the elevated/lowered left/right pelvic side in the frontal plane; shoulder rotation (SR) measures left/right (L/R) shoulder displacement in the transversal plane; pelvic rotation (PO) measures L/R rotation in the transversal plane; trochanter rotation (TR) measures L/R trochanter rotation in the transversal plane; condylus rotation (CR) measures knee rotation in the transversal plane; the sagittal distance of the cervical spine (SDCS) indicates the distance of the most protruded cervical (neck) vertebra in regards to the vertical line projection of the sacrum in the sagittal plane; the sag. sacrum/sagittal distance of the thoracic spine (SDTS) indicates the distance of the thoracic spine in regard to the vertical line projections of the sacrum in the sagittal plane; sag. distance lumbar spine–sacrum/sagittal distance of the lumbar spine (SDLS) indicates the distance of the lumbar (lower) spine in regards to the vertical line projection of the sacrum in the sagittal plane; varus/valgus left/X/O left leg (VVL) indicates the varus/valgus alignment angle of the left leg (medial/lateral) at the knee joint; varus/valgus right/X/O right leg (VVR) indicates the varus/valgus alignment angle of the right leg (medial/lateral) at the knee joint; flexion/extension left leg hyperextension/flexion of the left leg (FEL) indicates the hyperextension and flexion of the left leg at the knee joint in the sagittal plane; flexion/extension right leg (FER) indicates the hyperextension and flexion of the right leg at the knee joint in the sagittal plane; the frontal cervical spine (CS) indicates the distance of the cervical spine in the frontal plane in relation to the vertical line projection of the sacrum; the frontal thoracic spine (TS) indicates the distance of the thoracic spine in the frontal plane in relation to the vertical line projection of the sacrum; frontal lumbar spine (LS) indicates the distance of the lumbar spine in the frontal plane in relation to the vertical line projection of sacrum. These measurements were obtained using the 3D posture compact test protocol of the Contemplas measuring instrument [20]. The obtained parameters indicate possible deviations from zero (normal) values of the posture status in all three planes. A higher deviation value (positive or negative) implies a higher level of deformity [29].

### 2.3. Statistical Analysis

The data analysis was performed using SPSS V30.0 statistical software for the social sciences (SPSS Inc., Chicago, IL, USA). The following descriptive statistics were used to determine the basic descriptive parameters: mean and standard deviation (SD). The normality of data assessment was determined using the Kolmogorov–Smirnov test. As variables were not normally distributed, the nonparametric Mann–Whitney U test was used to determine differences between boys and girls in postural status. Cohen’s r value was used to measure the effect size with *r* ≥ 0.10 indicating a small effect; *r* ≥ 0.30 indicating a medium effect; and *r* ≥ 0.50 indicating a large effect [30,31,32]. There were no missing data. The Alfa level was set at *p* ≤ 0.05.

## 3. Results

Perusing the results of the posture recording by the Contemplas–Templo 3D compact mode procedure (Table 1), large variations in the angular parameters that describe the status of the legs in boys and girls are noticeable. A wide range of variable values show the status of the participants’ legs: varus/valgus left (VVL)/X/O left leg, varus/valgus right (VVR)/X/O right leg, flexion/extension left leg (FEL)/hyperextension/flexion left leg, and flexion/extension right leg (FER)/hyperextension/flexion right leg for relations between extension and flexion of the knee joint in the sagittal plane (legs are either slightly flexed or in hyperextension—sagittal plane, which shifts the angle ratios, e.g., from −163.18° to +174.27°). A similar finding is also present in the varus/valgus relation of the upper leg in relation to the lower leg (frontal plane of −173.97° to +179.55°). Such a range of results is determined by the position of the active, spherical benchmarks that represent the center of the hip joint—laterally, the center of the knee joint—laterally and the lateral malleolus, both for the right and for the left side (the position relationship of the mentioned benchmarks valorizes the angular values of the above variables). The position of the spherical markers is adjusted by software for 3D analysis and, in this case, reflects the state of a potential slight flexion or hyperextension in the knees in the upright position, recorded from the back for the variables flexion/extension left leg (FEL)/hyperextension/flexion of the left leg and flexion/extension right leg (FER)/hyperextension/flexion of the right leg. In addition, the spherical rappers reflect the potentially accentuated “Q” angle position of the center of the hip joint and the center of the knee joint, ending at the corresponding malleolus, in relation to the status of the subjects’ legs.

**Figure 2:**
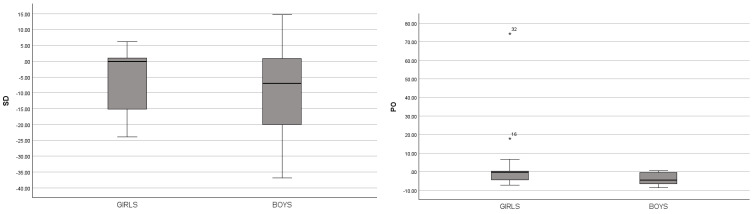

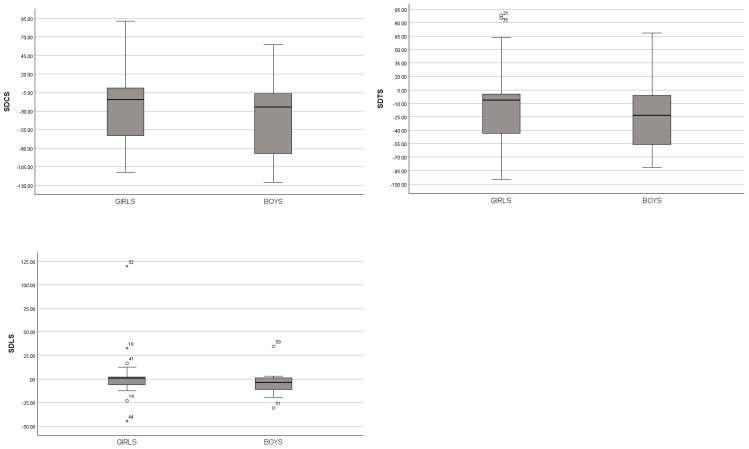
Boxplots of significant variables for boys and girls. * = an outlier.

By analyzing the results in Table 1, it was observed that boys exhibited significantly greater deviation from 0 in the following variables: shoulder displacement (SD) with *p* = 0.047; *r* = 0.21 (small effect), pelvic obliquity (PO) with *p* = 0.000; *r* = 0.37 (medium effect), sag. distance cervical spine–sacrum (SDCS) with *p* = 0.029; *r* = 0.23 (small effect), sag. distance thoracic spine–sacrum (SDTS) with *p* = 0.016; *r* = 0.25 (small effect), and sag. distance of the lumbar part of the spinal column (SDLS) with *p* = 0.005; *r* = 0.29 (small effect) (Figure 2). The effect size in the SD, SDCS, SDTS, and SDLS variables was small, while in the PO variable, it was considered medium, suggesting that the observed differences may have small to moderate clinical relevance.

## 4. Discussion

The present study highlighted gender differences in postural characteristics in preschool children. Boys showed a greater tendency towards postural deviations, indicating the necessity for specific interventions and programs to improve their posture.

Postural deformities in children have been the subject of numerous studies for many years [3,7,20,21,22,33,34,35]. The prevalence of postural deformities (30–80%) is very broad, reflecting variations in assessment methodologies, age groups, and definitions of postural abnormalities. A study on children aged 5–18 years [33] reported particularly high rates of scoliosis (86.5%), kyphosis (71%), and lordosis (28.4%), with 70% of participants exhibiting multiple disorders simultaneously. Similarly, research on school-aged children (6–15 years) [36] identified high frequencies of forward head posture (53.5%), shoulder elevation (74.3%), and winged scapulae (66.3%), highlighting the widespread nature of postural deviations. Beyond structural misalignments, the impact of body composition on postural health was evident in a study on children aged 3–18 years [37], where overweight and obese participants demonstrated significantly higher rates of postural errors (69.2% and 78.6%, respectively).

Large-scale research involving 595,057 Chinese children [34] found an overall prevalence of incorrect posture at 65.3%, with higher rates among older students and girls, suggesting a need for targeted intervention strategies. In younger populations, research on children aged 8–10 years [3] revealed that hyperlordosis (24.1%) was the most frequent condition, while scoliotic posture (33.3%) and flat back syndrome (18.4%) were also prevalent. Additionally, a study on children aged 6–8 years [35] reported fallen foot arches in 65% of participants, with 30% showing spinal curvature deformities in the sagittal plane and 13% in the frontal plane, reinforcing the interconnection between foot structure and spinal posture.

Further emphasizing the severity of postural issues, research conducted in Bulgaria on children aged 6–11 years [24] found incorrect posture in 58.85% of cases, while 23.67% were diagnosed with spinal deformities, raising concerns about the long-term implications of these conditions. Lastly, a study focusing on children aged 7–12 years [38] reported postural disorders in the sagittal plane in 83.9% of participants, indicating that spinal misalignments are highly prevalent in this age group.

Primary health care considers the prevention, early diagnosis, and treatment of postural deformities in children to be of great importance [16]. Poor posture in preschool children is an indicator of health problems that can become very serious if not corrected in time [15]. However, these issues are often not detected early enough. This underscores the important role educators play in supporting healthy growth and development. Through the regular monitoring and assessment of children’s posture, many health problems could be identified and addressed before they progress and leave serious consequences.

This research indicates that boys have more pronounced deformities in the following variables (Figure 2): shoulder displacement (SD) *p* = 0.047; *r* = 0.21, pelvic obliquity (PO) *p* = 0.000; *r* = 0.37, sag. distance cervical spine–sacrum (SDCS) *p* = 0.029; *r* = 0.23, sag. distance thoracic spine–sacrum (SDTS) *p* = 0.016; *r* = 0.016, and in sag. distance of the lumbar part of the spinal column (SDLS) *p* = 0.005; *r* = 0.29 compared to girls. The obtained values indicate statistically significant differences between the groups with small to medium effect sizes. Based on the results obtained, it is evident that boys have a more pronounced shoulder deviation (SD) from the ideal position compared to girls. Such a finding may indicate that growth patterns or developmental characteristics can influence the resulting postural differences, or that boys have certain differences in postural habits compared to girls. In Slovenian studies, postural deformities were found in one-half of the children. Deviations in the position of the shoulders and shoulder blades were recorded in more than 80% of children, while flat feet affected 65% of them [35].

Gender differences in this age group have been explored by researchers from several perspectives. The literature suggests that boys generally develop muscle strength later than girls, which potentially leads to weaker postural muscles during early childhood. This delayed development has been connected to higher incidences of postural issues, such as a winged scapula and shoulder imbalances, among boys, suggesting that scapular fixation occurs in boys at a later age [39]. The additional factor mentioned is flexibility. It has been highlighted that boys with limited flexibility exhibit more pronounced knee asymmetry and anteroposterior body tilt compared to girls, which contributes to a higher prevalence of postural deformities [40]. Research on posture gender differences, that is not directly connected to the same age group, suggests that the aforementioned increased flexibility in females might contribute to greater spinal, pelvic, and sacral mobility, which influences better posture maintenance in females [41]. Moreover, it has been noted that girls had better postural stability than boys, which may enable girls to maintain proper posture more effectively [42]. Additionally, the research has reported significant differences in male and female morphological characteristics in biceps and thigh and calf skin folds [12]. Furthermore, the results show that boys have a greater deformity compared to girls when it comes to the pelvic tilt (PO) variable. In Slovenian studies, pelvic anteversion and head protrusions were recorded in almost half of the cases [24]. Pelvic obliquity (PO) represents an abnormal position of the pelvic bone. This condition can lead to abnormal posture due to the compression and misalignment of the spine in order to compensate for any misalignment. The most common causes of an asymmetrical pelvis are uneven leg length, scoliosis of the spine, and muscle imbalances or contractures. These problems often occur in combination. Excessive sitting, poor posture, and muscle weakness can increase the risk of developing a tilted pelvis. A tilted or asymmetrical pelvis can also be the result of functional or structural problems. The most common structural causes are scoliosis and uneven leg lengths. Additionally, the variations in knee alignment observed in the sample (e.g., ‘X’ and ‘O’ leg positioning valorized by variables varus/valgus left (VVL)/X/O left leg, and varus/valgus right (VVR)/X/O right leg) suggest differing biomechanical adaptations in boys and girls. Knowing these facts, it can be determined that the results with large numerical ranges in the mentioned variables are adequate for clarifying the part of the investigation related to the postural qualities of the treated population in terms of potential leg deformities.

The obtained results for the variables sag. distance cervical spine–sacrum (SDCS) and sag. distance thoracic spine (SDTS) indicate a deviation from the physiological curves of the spinal column in the sagittal plane. The upright position of a person is conditioned by the continuous maintenance of balance between the paravertebral musculature and centripetal forces, as well as gravity. In the formation of the upright position in humans during evolution, physiological formations were created: lordosis in the cervical and lumbar parts of the spinal column, and kyphosis in the thoracic part. Within physiological limits, these curves are a normal phenomenon, while their increase or decrease is considered pathological [43]. It has been reported that deformities in the sagittal plane in first-year elementary school students can show up in 73.9% of the sample [44]. Excessive pelvic anteversion can lead to compensatory lumbar hyperlordosis, increasing mechanical stress on the lower back and predisposing individuals to chronic musculoskeletal discomfort. It was found that boys have a more frequent problem with cervical lordosis and with kyphotic body posture, as well as with a problem of holding the head, which should stand in the extension of the body, but which is actually moved forward. Boys’ posture is more often characterized by hyperkyphosis and lumbar hyperlordosis compared to girls [45,46]. The results of longitudinal studies indicate a higher incidence and increased tendency of boys toward the deformity of thoracic kyphosis [47]. Increased kyphosis and scoliosis, as compensatory postural outliers, in children have been connected to the increasingly greater lack of physical activity among children at this age [45].

The present study confirms the existence of significant sex differences in postural characteristics among preschool-aged children, with boys exhibiting more pronounced postural deviations in the sagittal plane, particularly in the shoulder and spinal regions. These findings indicate early manifestations of postural irregularities, underscoring the need for preventive interventions at this developmental stage. Similar patterns of postural deviations have been reported in previous studies that utilized the Contemplas 3D Posture Compact as a measurement instrument for posture analysis across the same and different age groups. Previous research has identified significant variations in postural parameters among children of different ages. One study [48] analyzed children aged 11–12 years and reported substantial differences in shoulder and pelvic rotation, sagittal distances, and flexion/extension parameters, confirming the high sensitivity of 3D posture assessment methods and highlighting the importance of early detection and prevention. Another study [21] investigated postural status in children aged 5–11 years and found considerable deviations in both the frontal and sagittal planes, including valgus knee alignment, knee joint hyperextension, and spinal asymmetry. Similarly, research on postural differences across multiple age groups (5–8, 9–11, and 12–14 years) [22] revealed a negative trend of increasing postural deformities associated with higher BMI, with boys showing a higher prevalence of thoracic kyphosis and shoulder girdle asymmetry, whereas girls exhibited a greater tendency toward lumbar lordosis and pelvic rotation. Additionally, a study focusing on children aged 6–9 years [29] confirmed sex-based differences in postural alignment, with girls being more prone to lumbar lordosis and valgus knee positioning, while boys demonstrated a higher occurrence of thoracic kyphosis. Moreover, an analysis of postural status in children aged 4–13 years [49] found that nearly 30% of participants had poor posture, with lower limb deformities being more common among girls. Overall, a vast majority of studies report significant differences between boys and girls while only one study we found reported no differences [44].

In line with these findings, the present study further supports the consistency of postural deviations identified using the Contemplas 3D Posture Compact system. By comparing our results with previous research, we observed that preschool-aged children exhibit similar patterns of postural misalignments to those reported in older age groups. This suggests that the early detection of postural irregularities using this technology can facilitate timely interventions aimed at mitigating progressive postural deformities. The reliability and precision of the Contemplas 3D Posture Compact system, as demonstrated across various studies, reinforce its applicability as a standardized tool for posture assessment in both research and clinical settings.

When assessing posture, X-ray imaging is still the gold standard [50]. However, optical analysis systems like the Contemplas Templo 3D have their own strengths and weaknesses. X-ray imaging has been reported to be particularly useful for detailed clinical skeletal assessments, making it an essential tool for diagnosing spinal conditions like scoliosis [51]. However, a major drawback is its exposure to ionizing radiation, which raises concerns about repeated use, particularly in children and young patients [52,53,54]. A noticeable limitation of X-rays is that they provide a static snapshot of posture, which may not fully capture functional posture changes/adaptations during movement [55,56]. Additionally, it is not readily accessible to clinicians [57]. On the other hand, photogrammetry with Contemplas Templo 3D and similar optical analysis systems are considered low-cost and completely non-invasive, making them safer for repeated use, especially for posture monitoring over time [58,59,60]. However, one drawback is their sensitivity to environmental conditions, such as lighting and camera positioning [61]. Additionally, having experienced raters can directly impact the reliability of clinical measures [62]. Therefore, having experienced raters with clinical and academic experience in postural assessment and human anatomy with regular online training is advisable to ensure high-quality posture analysis [57,62]. The literature suggests a two-level approach, where firstly, the photogrammetric methods should be used to assess the posture and, in case of concern, the second-level approach with radiography would be used, minimizing the need for repeated X-rays [63].

The modern way of life, especially hypokinesia, represents a real threat to maintaining a normal upright posture. It is assumed that the background of this body posture problem is most likely a result of daily irregular sitting for several hours [14], which leads to changes in the position of the head, thoracic and lumbar spine [64], inactivity of muscles responsible for maintaining correct upright posture, i.e., inactivity of the so-called antigravity musculature, which can lead to impaired bad postures, both at the level of the spinal column and lower extremities, incorrect position of the spine, weakness of the abdominal muscles, as well as the use of modern electronic devices [65,66]. Early intervention strategies, such as incorporating targeted postural exercises into school curricula, may help mitigate the impact of prolonged sitting and electronic device usage on musculoskeletal health [67,68,69]. Additionally, early screening is essential, and the usage of a photometric apparatus, like in the present study, which offers a fast and objective assessment of body posture, is advisable.

Postural deformities in children have become increasingly common. Rather than being primarily hereditary, these issues seem to nowadays largely stem from modern lifestyle changes affecting both children and their parents. Factors such as excessive screen time, sedentary behavior, and insufficient physical activity play a major role. Additionally, poor nutrition, a fast-paced and stressful lifestyle with limited time for healthy habits, and the rising prevalence of non-communicable diseases like obesity all contribute to the growing concern [70]. Additionally, the obtained results also emphasize the importance of education and intervention measures for correcting poor posture in children, considering that this can cause incurable damage to the musculoskeletal system, and neurological and pathological damage in the future. Moreover, studies have suggested that specially programmed and planned exercises and physical activity can substantially contribute to the development of good posture and should be viewed as an important factor of prevention in the daily activities of children [23,24,71,72]. Research has shown that specific structured movement activities in youth ages may be more effective in the development of motor coordination, agility, and speed of movement in children [73].

Based on the results of this research, several directions for future research and application in practice are recommended:

- Conducting cross-cultural research that would enable the comparison of results among children from different environments and cultural contexts, which would determine eventual differences and specificities in the development of postural characteristics.
- Including a larger sample of participants in future research, including children from rural and urban areas, to ensure the representativeness and generalizability of the results.
- Conducting a longitudinal study that would follow the postural characteristics of children through different developmental stages, aiming to identify critical periods for intervention and determine more precisely developmental trends in the context of gender differences.
- Conducting research with a larger sample of subjects and by including factors of physical activity and lateralization could provide more detailed insights into these variables and enable a better understanding of their influence on postural status.

The present study needs to acknowledge some limitations. One of them is that the participants’ physical activity was not monitored, nor was their body lateralization analyzed. The lack of the analysis of these factors can limit the interpretation of the results because physical activities and lateralization can significantly impact postural status and the development of deformities. Therefore, further studies should account for that. The obtained results indicate statistically significant differences between the recorded groups. Although the effect size of these differences is small, these asymmetries should be interpreted carefully. Additionally, a larger sample size would be beneficial.

## 5. Conclusions

The results of this research indicate that boys have more pronounced postural deformities than girls, which is especially evident in variables such as shoulder displacement, pelvic tilt, and sagittal deviations in the cervical and thoracic spine. For early screening, the usage of a photometric apparatus that offers a fast and objective assessment of body posture is advisable. The differences in posture between boys and girls may be influenced by their physical development, daily habits, and the types of activities they engage in. Factors such as growth patterns, muscle balance, and lifestyle choices—like prolonged sitting and the frequent use of electronic devices—could contribute to postural issues. To better understand these differences, future research should explore how posture develops over time in larger, more diverse groups. Cross-cultural studies and investigations into physical activity and movement preferences could provide valuable insights into the key factors shaping children’s posture.

## Figures and Tables

**Table 1:** A table containing descriptive statistics of selected variables, Mann–Whitney U test between genders, and effect size.

Variables	Girls		Boys		Mean Rank		U	Z	*p*	*r*
	Mean	SDv	Mean	SDv	Girls	Boys				
SD (cm)	−4.7	8.78	−9.94	12.32	52.0	41.0	803.5	−1.99	0.047 *	0.21
PO (cm)	0.49	11.8	−3.62	3.06	56.4	36.6	603.5	−3.55	0.000 *	0.37
SR (°)	26.95	42.4	32.6	46.13	44.8	48.2	981.5	−0.60	0.550	0.06
PR (°)	26.6	45.9	27.26	53.88	47.9	45.1	994.5	−0.50	0.620	0.05
TR (°)	40.87	47.4	49.99	58.69	43.9	49.1	937.5	−0.94	0.347	0.10
CR (°)	4.17	55.1	16.84	73.68	42.1	50.9	857.5	−1.57	0.117	0.16
SDCS (cm)	−18.1	45.7	−44.7	48.98	52.6	40.4	778.5	−2.18	0.029 *	0.23
SDTS (cm)	−8.67	37.9	−27.8	33.21	53.2	39.8	748.5	−2.42	0.016 *	0.25
SDLS (cm)	1.75	20.7	−4.85	9.69	54.3	38.7	699.5	−2.80	0.005 *	0.29
VVL (°)	10.47	84.3	22.12	62.22	41.9	51.1	848.5	−1.64	0.102	0.17
VVR (°)	−27.1	92	−46.1	101.1	48.0	45.0	987.5	−0.55	0.582	0.06
FEL (°)	5.34	74.1	−14.8	68.07	47.5	45.5	1014	−0.35	0.728	0.04
FER (°)	−36.1	91.7	−53.3	101.2	49.3	43.7	927.5	−1.02	0.308	0.11
CS (cm)	0.22	14.7	−0.59	27.89	48.3	44.7	975.5	−0.64	0.519	0.07
TS (cm)	2.4	11.5	2.99	26.67	49.5	43.5	921.5	−1.07	0.286	0.11
LS (cm)	1.14	7.13	2.97	14.62	49.4	43.6	926	−1.03	0.303	0.11

Legend: All values are presented as mean ± standard deviation. Variables marked with * indicate statistically significant differences (*p* ≤ 0.05); standard deviation (SDv); shoulder displacement (SD); pelvic obliquity (PO); shoulder rotation (SR); pelvic rotation (PO); trochanter rotation (TR); condylus rotation/knee rotation (CR); sag. distance cervical spine–sacrum/sagittal distance of the neck part of the spinal column (SDCS); sag. distance thoracic spine–sacrum/sagittal distance of the thoracic spine (SDTS); sag. distance lumbar spine–sacrum/sagittal distance of the lumbar part of the spinal column (SDLS); varus/valgus left/X/O left leg (VVL); varus/valgus right/X/O right leg (VVR); flexion/extension left leg hyperextension/flexion of the left leg (FEL); flexion/extension right leg/hyperextension of the right leg (FER); frontal cervical spine/frontal distance of the neck part of the spinal column (CS); frontal thoracic spine/frontal distance of the chest part of the spinal column (TS); frontal lumbar spine/frontal distance of the lumbar part of the spinal column (LS); Mann–Whitney U test statistic (U); Mann–Whitney U test Z statistic (Z); Mann–Whitney U test significance value (*p*); Cohen’s r effect size (*r*).

## Data Availability

The data supporting this study’s findings are available from the corresponding author upon reasonable request.

## References

[1] Skender N., Pistotnik B., Čolakhodžić E. (2007). Basics of Movement in Sports.

[2] Augustinović I., Halilović S., Dobrić A., Kovačevska K., Kovač L., Matošević L., Kosterović A., Begović H., Jakešević B., Barnjak T. (2018). The Present Development Program of Pre-School Upbringing and Education of the Canton of Central Bosnia.

[3] Baranowska A., Sierakowska M., Owczarczuk A., Olejnik B.J., Lankau A., Baranowski P. (2023). An Analysis of the Risk Factors for Postural Defects among Early School-Aged Children. J. Clin. Med..

[4] Plandowska M., Lichota M., Górniak K. (2019). Postural Stability of 5-Year-Old Girls and Boys with Different Body Heights. PLoS ONE.

[5] Peulić J., Katanić B., Jovanović N., Bjelica D. (2024). Exploring Anthropometric Characteristics, Weight Status, and Posture among Preschool Children in Serbia. J. Anthr. Sport Phys. Educ..

[6] Rusek W., Baran J., Leszczak J., Adamczyk M., Baran R., Weres A., Inglot G., Czenczek-Lewandowska E., Pop T. (2021). Changes in Children’s Body Composition and Posture during Puberty Growth. Children.

[7] Vranešić-Hadžimehmedović D., Arapović M., Hodžić A., Mašić S. (2024). Prevalence of spinal deformities in preschool children living in the SOS children’s village in Sarajevo. Homosporticus.

[8] Standring S. (2005). Gray’s Anatomy: The Anatomical Basis of Clinical Practice.

[9] Protić-Gava B., Šćepanović T. (2016). Basis of Kinesitherapy and Applied Corrective Gymnastic.

[10] Madić D. (2014). Improving Testing Abilities on Postural and Spinal Column Status—Spinelab.

[11] Čović N. (2020). The Influence of a Program of Motor Control Exercises on Body Composition, Postural Status and Motor Skills in Young Athletes. Ph.D. Thesis.

[12] McEvoy M.P., Grimmer K. (2005). Reliability of Upright Posture Measurements in Primary School Children. Biomed. Cent. Ser. Musculoskelet. Disord..

[13] Romanov R., Stupar D., Međedović B., Brkin D. (2014). Postural Status of Preschool Children in the Territory of Novi Sad. TIMS Acta.

[14] Sabo E. (2006). Postural Status of Preschool Children in Novi Sad. Pedagog. Real..

[15] Džibrić D. (2019). Differences in Morphological and Postural Status between Primary School Males and Females. Sport Sci. Pract. Asp..

[16] Kolybacz A., Niewiem M., Buczyńska A., Woś H. (2019). Health Status of 9-Year-Old Children in Katowice. Stand. With. Pediatr..

[17] Glavaš J., Rumboldt M., Karin Ž., Matković R., Kresina S., Dragaš-Zubalj N., Aljinović J. (2023). The role of school medicine in the early detection and management of adolescent idiopathic scoliosis. Wien. Klin. Wochenschr..

[18] Rusnák R., Kolarová M., Aštaryová I., Kutiš P. (2019). Screening and Early Identification of Spinal Deformities and Posture in 311 Children: Results from 16 Districts in Slovakia. Rehabil. Res. Pract..

[19] Miletić A., Protić-Gava B. (2017). Gender-Related Morphological Characteristics in Preschool Children of Kolubara District. EQOL J..

[20] Kovač S., Kajmović H., Rađo I., Manić G. (2014). Trend Projections of Body Deformities Occurrence between the Ages of 5 and 12, Metrically Objectified and Estimated by 3D Postural Status Screening. Sporty Man.

[21] Kovač S., Kapo S., Alić H., Manić G. (2015). Postural Quality Analyses for Children Recorded in Kinematic 2D and 3D Contemplas Method. Tech. Technol. Educ. Manag..

[22] Kapo S., Rađo I., Smajlović N., Kovač R., Talović M., Doder L., Čović N. (2018). Increasing Postural Deformity Trends and Body Mass Index Analysis in School-Age Children. Slov. J. Public Health.

[23] Bićanin P., Milenković S., Radovanović D., Gajević A., Ivanović J. (2017). Postural Disorders in Preschool Children in Relation to Gender. Univ. Facts Phys. Educ. Sports.

[24] Mitova S. (2015). Frequency and Prevalence of the Postural Disorders and Spinal Deformities in the Children of Primary School Age. Res. Kinesiol..

[25] Faul F., Erdfelder E., Buchner A., Lang A.G. (2009). Statistical Power Analyses Using G*Power 3.1: Tests for Correlation and Regression Analyses. Behav. Res. Methods.

[26] Tanita Corporation (2015). BC-420MA Body Composition Monitor Instruction Manual.

[27] Widhalm K., Durstberger S., Greisberger A., Wolf B., Putz P. (2024). Validity of Assessing Level Walking with the 2D Motion Analysis Software TEMPLO and Reliability of 3D Marker Application. Sci. Rep..

[28] Šćepanović T., Marinković D., Korovljev D., Madić D. The Status of the Spine in the Sagittal Plane in Girls. Proceedings of the 5th International Scientific Conference Contemporary Kinesiology.

[29] Ćirić A., Čaušević D., Bejdić A. (2015). Differences in Postural Status between Boys and Girls Aged 6–9 Years Recorded with the 3D Method of the Measuring Instrument Contemplas. Homosporticus.

[30] Cohen J. (1988). Statistical Power Analysis for the Behavioral Sciences.

[31] Fritz C.O., Morris P.E., Richler J.J. (2012). Effect Size Estimates: Current Use, Calculations, and Interpretation. J. Exp. Psychol. Gen..

[32] Tomczak M., Tomczak E. (2014). The Need to Report Effect Size Estimates Revisited: An Overview of Some Recommended Measures of Effect Size. Trends Sport Sci..

[33] Dop D., Pădureanu V., Pădureanu R., Niculescu S.-A., Drăgoescu A.N., Moroșanu A., Mateescu D., Niculescu C.E., Marcu I.R. (2024). Risk Factors Involved in Postural Disorders in Children and Adolescents. Life.

[34] Yang L., Lu X., Yan B., Huang Y. (2020). Prevalence of Incorrect Posture among Children and Adolescents: Finding from a Large Population-Based Study in China. iScience..

[35] Kolarova M., Kutiš P., Rusnak R., Hrčková Z., Hudáková Z., Lysá L., Luliak M., Babel’a R. (2019). Analysis of Body Segments and Postural State in School Children. Neuroendocrinol. Lett..

[36] Batistão M.V., Carreira Moreira R.F., Cote Gil Coury H.J., Salasar L.E.B., Sato T.O. (2016). Prevalence of Postural Deviations and Associated Factors in Children and Adolescents: A Cross-Sectional Study. Fisioter. Mov..

[37] Maciałczyk-Paprocka K., Stawińska-Witoszyńska B., Kotwicki T., Sowińska A., Krzyżaniak A., Walkowiak J., Krzywińska-Wiewiorowska M. (2017). Prevalence of incorrect body posture in children and adolescents with overweight and obesity. Eur. J. Pediatr..

[38] Bubanj S., Đorđević S., Milenković S., Stanković R., Vidojević M., Đokić M. (2021). Postural Disorders and Muscle Power in Primary School Children. Acta Fac. Medicae Naissensis.

[39] Penha P.J., Casarotto R.A., Sacco I.C.N., Marques A.P., João S.M.A. (2008). Qualitative Postural Analysis among Boys and Girls of Seven to Ten Years of Age. Braz. J. Phys. Ther..

[40] Coelho J.J., Graciosa M.D., de Medeiros D.L., Pacheco S.C., da Costa L.M., Ries L.G. (2014). Influence of Flexibility and Gender on the Posture of School Children. Rev. Paul. Pediatr..

[41] Mizukoshi R., Yagi M., Yamada Y., Yokoyama Y., Yamada M., Watanabe K., Nakamura M., Nagura T., Jinzaki M. (2024). Gender Differences in Spinal Mobility During Postural Changes: A Detailed Analysis Using Upright CT. Sci. Rep..

[42] Smith A.W., Ulmer F.F., Wong D.P. (2012). Gender Differences in Postural Stability Among Children. J. Hum. Kinet..

[43] Gajić D. (2009). Kinesitherapy in Spine Deformity. Master’s Thesis.

[44] Đorđević S., Jorgić B., Milenković S., Stanković R., Stanković M. (2020). The Incidence of Spinal Postural Disorders in First-Grade Elementary-School Students. Facta Univ. Ser. Phys. Educ. Sport.

[45] Đorđević S., Stanković M., Jorgić B., Milenković S., Smailović S., Katanić B., Jelaska I., Pezelj L. (2024). The Association of Sagittal Spinal Posture among Elementary School Pupils with Sex and Grade. Children.

[46] Wojtków M., Szkoda-Poliszuk K., Szotek S. (2018). Influence of Body Posture on Foot Load Distribution in Young School-Age Children. Acta Bioeng. Biomech..

[47] Poussa M.S., Heliövaara M.M., Seitsamo J.T., Könönen M.H., Hurmerinta K.A., Nissinen M.J. (2005). Development of Spinal Posture in a Cohort of Children from the Age of 11 to 22 Years. Eur. Spine J..

[48] Skender N., Kurtović N., Kovač S., Šabić E., Ćeleš N. (2023). Two Different Methods Comparative Analysis of Determining Body Deformities in Students. Homosporticus.

[49] Kojić M. (2014). Differences in Indicators of Postural Status between Boys and Girls from Srem. Exerc. Qual. Life.

[50] Ng S.Y., Bettany-Saltikov J. (2017). Imaging in the Diagnosis and Monitoring of Children with Idiopathic Scoliosis. Open Orthop. J..

[51] Weigel S., Dullien S., Grifka J., Jansen P. (2024). Comparison Between Rasterstereographic Scan and Orthopedic Examination for Posture Assessment: An Observational Study. Front. Surg..

[52] Kutanzi K.R., Lumen A., Koturbash I., Miousse I.R. (2016). Pediatric Exposures to Ionizing Radiation: Carcinogenic Considerations. Int. J. Environ. Res. Public Health.

[53] Inoue D., Shigematsu H., Nakagawa Y., Takeshima T., Tanaka Y. (2021). The Influence of Posture on Instability Evaluation Using Flexion-Extension X-Ray Imaging in Lumbar Spondylolisthesis. Asian Spine J..

[54] Dorfman A.L., Fazel R., Einstein A.J., Applegate K.E., Krumholz H.M., Wang Y., Christodoulou E., Chen J., Sanchez R., Nallamothu B.K. (2011). Use of Medical Imaging Procedures with Ionizing Radiation in Children: A Population-Based Study. Arch. Pediatr. Adolesc. Med..

[55] Dreischarf M., Pries E., Bashkuev M., Putzier M., Schmidt H. (2016). Differences Between Clinical “Snap-Shot” and “Real-Life” Assessments of Lumbar Spine Alignment and Motion—What Is the “Real” Lumbar Lordosis of a Human Being?. J. Biomech..

[56] Sugiyama N., Kai Y., Koda H., Morihara T., Kida N. (2024). Agreement in the Postural Assessment of Older Adults by Physical Therapists Using Clinical and Imaging Methods. Geriatrics.

[57] Hébert-Losier K., Abd Rahman F. (2017). Reliability of postural measures in elite badminton players using Posture Pro 8. Physiother. Theory Pract..

[58] Van Niekerk S.M., Louw Q., Vaughan C., Grimmer-Somers K., Schreve K. (2008). Photographic measurement of upper-body sitting posture of high school students: A reliability and validity study. BMC Musculoskelet. Disord..

[59] Ruivo R.M., Pezarat-Correia P., Carita A.I., Vaz J.R. (2013). Reliability and validity of angular measures through the software for postural assessment. Rehabilitación.

[60] Ha S.M., Kwon O.Y., Weon J.H., Kim M.H., Kim S.J. (2013). Reliability and validity of goniometric and photographic measurements of clavicular tilt angle. Man. Ther..

[61] Alam M.F., Zaki S., Sharma S., Nuhmani S. (2024). Establishing the Reliability of the GaitON Motion Analysis System: A Foundational Study for Gait and Posture Analysis in a Healthy Population. Sensors.

[62] Ludwig O., Hammes A., Kelm J., Schmitt E. (2016). Assessment of the posture of adolescents in everyday clinical practice: Intra-rater and inter-rater reliability and validity of a posture index. J. Bodyw. Mov. Ther..

[63] Roggio F., Ravalli S., Maugeri G., Bianco A., Palma A., Di Rosa M., Musumeci G. (2021). Technological Advancements in the Analysis of Human Motion and Posture Management through Digital Devices. World J. Orthop..

[64] Claus A.P., Hides J.A., Moseley G.L., Hodges P.W. (2016). Thoracic and Lumbar Posture Behaviour in Sitting Tasks and Standing: Progressing the Biomechanics from Observations to Measurements. Appl. Ergon..

[65] Straker L.M., O’Sullivan P.B., Smith A., Perry M. (2007). Computer Use and Habitual Spinal Posture in Australian Adolescents. Public Health Rep..

[66] Simov S.B., Milinić S.M., Stojanović D.O. (2011). Frequency of Poor Posture and Flat Feet in Preschool Children. Apollo Med. Aescul..

[67] Slováková M., Mandzáková M. (2024). Effect of an exercise program on the body posture of young school-aged pupils. J. Phys. Educ. Sport.

[68] Araújo C.L., Moreira A., Carvalho G.S. (2023). Postural Education Programmes with School Children: A Scoping Review. Sustainability.

[69] Miñana-Signes V., Monfort-Pañego M., Valiente J. (2021). Teaching Back Health in the School Setting: A Systematic Review of Randomized Controlled Trials. Int. J. Environ. Res. Public Health.

[70] Lopez-Fuenzalida A., Rodriguez Channels C., Reyes Ponce A., Contreras Molina A., Quezada J.F., Polanco C.A. (2016). Association between Nutritional Status and Flat Foot Prevalence in Chilean Children from 6 to 10 Years of Age. Nutr. Hosp..

[71] Fernandes O., Basilio F. (2016). Tai Chi training can improve postural control. Rev. Artes Marciales Asiáticas.

[72] Wąsik J., Motow-Czyz M. (2015). Comparative analysis of body posture in child and adolescent taekwon-do practitioners and non-practitioners. Ido Mov. Cult. J. Martial Arts Anthropol..

[73] Kojić F., Arsenijević R., Grujić G., Toskić L., Šimenko J. (2024). Effects of Structured Physical Activity on Motor Fitness in Preschool Children. Children.

